# Clustering single-cell multi-omics data with MoClust

**DOI:** 10.1093/bioinformatics/btac736

**Published:** 2022-11-16

**Authors:** Musu Yuan, Liang Chen, Minghua Deng

**Affiliations:** Center for Quantitative Biology, Peking University, Beijing 100871, China; Huawei Technologies Co., Ltd., Beijing 100080, China; Center for Quantitative Biology, Peking University, Beijing 100871, China; School of Mathematical Sciences, Peking University, Beijing 100871, China; Center for Statistical Science, Peking University, Beijing 100871, China

## Abstract

**Motivation:**

Single-cell multi-omics sequencing techniques have rapidly developed in the past few years. Clustering analysis with single-cell multi-omics data may give us novel perspectives to dissect cellular heterogeneity. However, multi-omics data have the properties of inherited large dimension, high sparsity and existence of doublets. Moreover, representations of different omics from even the same cell follow diverse distributions. Without proper distribution alignment techniques, clustering methods will encounter less separable clusters easily affected by less informative omics data.

**Results:**

We developed MoClust, a novel joint clustering framework that can be applied to several types of single-cell multi-omics data. A selective automatic doublet detection module that can identify and filter out doublets is introduced in the pretraining stage to improve data quality. Omics-specific autoencoders are introduced to characterize the multi-omics data. A contrastive learning way of distribution alignment is adopted to adaptively fuse omics representations into an omics-invariant representation. This novel way of alignment boosts the compactness and separableness of clusters, while accurately weighting the contribution of each omics to the clustering object. Extensive experiments, over both simulated and real multi-omics datasets, demonstrated the powerful alignment, doublet detection and clustering ability features of MoClust.

**Availability and implementation:**

An implementation of MoClust is available from https://doi.org/10.5281/zenodo.7306504.

**Supplementary information:**

Supplementary data are available at *Bioinformatics* online.

## 1 Introduction

The recent technological innovation of single-cell genomics allows for assaying cellular heterogeneity at an unprecedented resolution. It provides us an unparalleled opportunity to dissect complex biological processes, including cancer (Bian *et al.*, 2018) and embryonic development. Recently, several single-cell multi-omics technologies have merged, allowing multiple biological layers to be probed in parallel in the same cell. Representative methods include single-cell Cellular Indexing of Transcriptomes and Epitopes (CITE-seq) (Stoeckius *et al.*, 2017), single-cell DNA methylation and transcriptome (scM&T-seq) (Angermueller *et al.*, 2016), single-nucleus chromatin accessibility and mRNA expression sequencing (SNARE-seq) (Chen *et al.*, 2019) and single-cell gDNA-mRNA sequencing (DR-seq) (Dey *et al.*, 2015). We segmented these technologies according to omics in Figure 2a. In single-omics data analysis, a crucial step involves clustering cells into subpopulations to facilitate subsequent downstream analysis. Emerging single-cell multi-omics data can potentially enrich cell type-specific information across different omics, yet clustering methods still need to be tailored to fully utilize these abundant, yet complex, datasets.

Single-cell multi-omics data inherit the problems existing in scRNA-seq and other single-omics data, such as large dimensions, high sparsity and high noise. These barriers make it very difficult to characterize single-cell multi-omics data and effectively extract cell type-specific informations. Moreover, single-cell multi-omics data often contain a fair proportion of doublets which are pseudo counts that typically arise owing to errors in cell sorting or capture (Kim *et al.*, 2020). Most existing methods detect doublets by generating artificial doublets, evaluating the similarities between samples and the artificial doublets then manually setting a threshold for the doublet score (Xi and Li, 2020). However, true doublets are not necessarily similar to artificial doublet, and we need to separate those generated doublets far from normal droplets. Additionally, without knowing the proportion of doublets, the threshold for doublet scoring is hard to select.

**Fig. 1.**
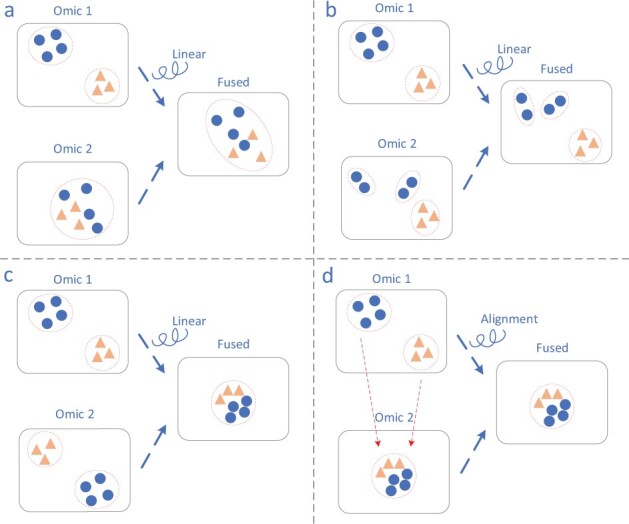
Linear fusion may hamper clustering. (**a**) Without alignment, less informative omics data make the fused clusters less separable. (**b**) Without alignment, less informative omics data make fused clusters less compact. (**c**) Without alignment, accurate clusters are attainable with each omics data, but the combination may be of poor quality. (**d**) Existing one-to-one cluster alignment fails when one of multiple omics is indistinguishable

Other difficulties also arose when clustering single-cell multi-omics data. The first involves large differences in dimension among various omics data, making it difficult to extract omics-invariant data which benefits clustering in the same manner from each omics. Second, different omics data distribute divergently, even in the same cell, and omics are not necessarily equally important to the clustering objective. Linearly combining different omics representations is the most popular way of handling multi-omics data. However, without proper distribution alignment methods, the contribution of each omics is hard to assess, and the compactness and separability of clusters may also be harmed. To be more intuitive, we depicted in Figure 1 several cases in which linear combination will hamper the clustering analysis. When some omics data are not informative enough to obtain accurate clusters, the linear combination will encounter less separable or compact clusters. Even when accurate clusters are available with every single-omic data, the relative positions among clusters vary dramatically, potentially making the fused data less suitable for clustering. Existing alignment methods are sometimes subject to pitfalls. Suppose that no clusters can be distinguished by the representation of an individual omics. In this extreme case, all other omics will be aligned to it, resulting in a fully inseparable fused representation. Also, since the multi-omics data have a larger scale than most of single-omics data, clustering methods have to be more scalable than ever to avoid excessive computing resource and time consumption.

**Fig. 2.**
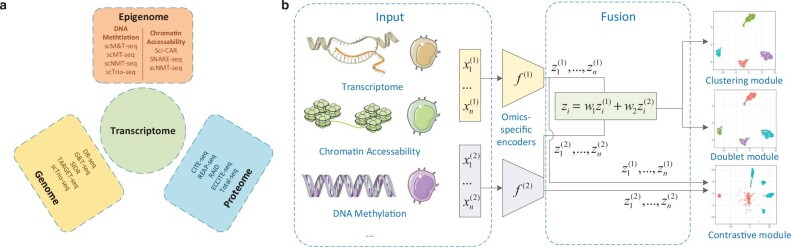
Framework overview. (**a**) Existing multi-omics sequencing methods, grouped by different omics they can sequence. (**b**) Structure of the MoClust model: (i) preprocessed multi-omics data (Cao and Gao, 2022) are used as input, while outputs are estimated posterior parameters of omics-specific statistical models (Section 2.1). (ii) A fusion layer is introduced to linearly fuse the latent features of different omics data and is guided by a contrastive learning module (Section 2.2). (iii) A Cauchy–Schwarz divergence-based clustering module (Section 2.3) and a novel automatic doublet detection module (Section 2.4) are added after the fusion layer

Recently, several computational methods able to cluster single-cell multi-omics data have been developed. MOFA+ (Argelaguet *et al.*, 2020) is the sequel to MOFA (Multi-Omics Factor Analysis) (Argelaguet *et al.*, 2018). Both perform factor analysis, learning reduced and common features (‘factors’) to capture omics-invariant information. Clustering is conducted by k-means algorithm using the factor matrix. MOFA+ scales easily to large datasets and is widely used by the community, but it weights each omics as equally important in the optimization term and is thus easily affected by the less informative omics. Additionally, MOFA+ suffer from the poor ability of Principal Component Analysis (PCA) to capture complex nonlinear correlations in single-cell multi-omics data. Other methods utilizing linear dimension reduction techniques also endure this problem, including Seurat (Hao *et al.*, 2021), Liger (Liu *et al.*, 2020) and Harmony (Korsunsky *et al.*, 2019).

On the other hand, deep generative models have been introduced into single-cell multi-omics data clustering analysis and have quickly gained popularity. TotalVI (Gayoso *et al.*, 2021) and MultiVI utilize variational autoencoders (VAE) to handle different types of single-cell multi-omics data. They average the low-dimensional representations learned by omics-specific encoders and conduct Leiden algorithm to obtain clusters. Since the clustering stages are separated from dimension reductions, the latent space may not be well-suited for clustering. Additionally, they are not equipped with distribution alignment modules and treat different omics equivalently, thus suffer the same problems as MOFA+ does. Deep cross-omics cycle attention (DCCA) model (Zuo *et al.*, 2021) is another VAE-based method, but employing an attention-transfer technique (Vaswani *et al.*, 2017) to perform a cluster-to-cluster alignment between latent omics representations. However, one-to-one alignments of clusters are only attainable when encoders can separate all clusters in all omics, which is not possible in practice. Additionally, the iterations of teach-feedback loop need to be tuned elaborately.

More and more clustering methods for single-cell multi-omics data attach importance to representation alignment (Trosten *et al.*, 2021) between different omics. Proper alignment techniques help in effectively weighting the contributions of different omics to the clustering objective. It can also enrich cell type-specific information and make the clusters more separable and compact. However, the most popular attention-based alignment methods require full separability of all omics representations, and their performance deteriorates rapidly when the quality of a single-omics worsens. For example, when clustering transcriptomic and epigenomic data, it is inevitable that one omics (epigenome) is much less informative than another (transcriptome). This calls for an improved representation alignment method. Fortunately, contrastive learning (Chen *et al.*, 2020) offers a novel way to leverage the advantages of alignment, preserving local geometric structures and omics-invariance, while having fewer constraints on separability of each omics. Instead of the current one-to-one alignment of clusters, the contrastive method can operate alignment at the sample level and effectively alleviate the problems suffered by cluster-level alignment. The self-supervised contrastive model has proven to be very powerful in various computer vision tasks, but it still has not been introduced into single-cell multi-omics integration analysis.

Here, we introduce MoClust as a model for clustering single-cell multi-omics data. MoClust can be applied to transcriptomic and proteomic data, as well as transcriptomic and epigenomic data. The main idea of it is illustrated in Figure 2b. First, it separately models each omics dataset by different autoencoders and learns low-dimensional omics-specific latent representations. Second, in the pretraining stage, a novel automatic doublet discovery module is introduced to purify input data and boost clustering performance. Third, MoClust contains a contrastive module for representation alignment. Similarities between representations are estimated by cosine angles rather than Euclidean distances to adjust the potential inconsistent metrics in different omics. The positive pairs are selected as different omics representations of the same cell and negative pairs are randomly sampled cell representations with distinct predicted clusters. This pairing policy assures that MoClust aligns representations at the sample level, avoiding the problem of misaligned label distributions. Adaptive weights learned by this contrastive module are utilized to fuse latent representations. Finally, a Cauchy–Schwarz divergence (CS divergence)-based (Jenssen *et al.*, 2006) clustering is conducted with the fused representation. This novel clustering module promotes the compactness and separability of clusters, while pushing the clustering assignment vectors toward orthogonality and approaches toward simplex. To demonstrate the efficiency of MoClust, we applied it and other state-of-the-art clustering methods to both real and simulated datasets, including CITE-seq and SNARE-seq datasets. Through extensive evaluations, we demonstrated that MoClust has excellent and unbiased performance on clustering over all cases.

## 2 Materials and methods

Suppose our dataset consists of *n* samples (cells) observed in *M* different omics. Let $x_{i}^{\left(m\right)}$ be the observation of sample *i* from omic *m*, then the total observation of cell *i* is $x_{i}^{1},\ldots ,x_{i}^{\left(M\right)}$. Given cluster (cell type) number *K*, our objective is to assign the observation set of each cell *i*, $x_{i}^{1},\ldots ,x_{i}^{\left(M\right)}$, to one of the *K* clusters. For protein data preprocessing, we normalize the counts for each cell after log transforming and finally scale the data. For RNA-seq data, we do the same preprocess and compute the size factor for each cell. For ATAC-seq data, we first generate a rough estimate of the transcriptional activity of each gene by quantifying ATAC-seq counts in the 2 kb-upstream region and gene body. And then we treat the gene activity matrix as RNA-seq data. The raw counts of RNA data and gene activity matrix will also be fed to the model to compute the reconstruction, i.e. NB(ZINB), loss.

### 2.1 ZINB model-based autoencoder

First, we transform each preprocessed $x_{i}^{\left(m\right)}$ to its low-dimensional representation $z_{i}^{\left(m\right)}$ with an omic-specific encoder network $f^{\left(m\right)}$

(1) $$z_{i}^{\left(m\right)}=f^{\left(m\right)}(x_{i}^{\left(m\right)})$$

To capture the character of transcriptomic (scRNA-seq) and epigenomic data, we utilize the same ZINB (Eraslan *et al.*, 2019) model-based autoencoder as in scSemiCluster (Chen *et al.*, 2021). Protein data are not as sparse as those in the other two, therefore, we empirically use a negative binomial (NB) model, a ZINB model hybrid, to characterize it. We modeled gene activity matrices converted from ATAC-seq data drawn from ZINB as well. Formally, ZINB is parameterized with mean (μ) and dispersion (θ) of the negative binomial distribution and with an additional coefficient (*π*) that represents the weight of the point mass of probability at zero (the probability of dropout events):

(2) $$\begin{array}{c} \text{NB}\left(X_{\mathrm{raw}}^{\left(m\right)}|\mu^{\left(m\right)},\theta^{\left(m\right)}\right)=\frac{\Gamma \left(X_{\mathrm{raw}}^{\left(m\right)}+\theta^{\left(m\right)}\right)}{\Gamma (X_{\mathrm{raw}}^{\left(m\right)}+1)\Gamma (\theta^{\left(m\right)})}\\ \times {\left(\frac{\theta^{\left(m\right)}}{\theta^{\left(m\right)}+\mu^{\left(m\right)}}\right)}^{\theta^{\left(m\right)}}{\left(\frac{\mu^{\left(m\right)}}{\theta^{\left(m\right)}+\mu^{\left(m\right)}}\right)}^{X_{\mathrm{raw}}^{\left(m\right)}}\\ \;\text{ZINB}\;\left(X_{\mathrm{raw}}^{\left(m\right)}|\pi^{\left(m\right)},\mu^{\left(m\right)},\theta^{\left(m\right)}\right)=\pi^{\left(m\right)}G_{0}\left(X_{\mathrm{raw}}^{\left(m\right)}\right)\\ +(1-\pi^{\left(m\right)})\text{NB}\left(X_{\mathrm{raw}}^{\left(m\right)}|\mu^{\left(m\right)},\theta^{\left(m\right)}\right) \end{array}$$

where $X_{\mathrm{raw}}^{\left(m\right)}$ represents the raw read counts from omics *m*. The ZINB model-based autoencoder for *m*-th omics estimates the parameters $\mu^{\left(m\right)},\theta^{\left(m\right)}$ and $\pi^{\left(m\right)}$ by constructing three parallel output layers. The loss function of the ZINB model-based autoencoder is the negative log-likelihood of ZINB distribution:

(3) $$L_{\text{ZINB}}=-\sum_{m\in M_{Z}}\gamma_{m}\;\text{log}\;\left(\text{ZINB}\left(X_{\mathrm{raw}}^{\left(m\right)}|\mu^{\left(m\right)},\pi^{\left(m\right)},\theta^{\left(m\right)}\right)\right)$$

where $M_{Z}$ is the set of different omics and *γ_m_* refers to the strength of ZINB loss for the *m*-th omics. We introduce a fusion layer to represent the integrated information of all different omics, which computes a weight average

(4) $$z_{i}=\sum_{m=1}^{M}w_{m}z_{i}^{\left(m\right)}$$

where *w_m_* are fusion weights which are contributions of omics to the omic-invariant representation *z_i_*. These weights will be adaptively chosen under the guidance of the following contrastive alignment.

### 2.2 Contrastive alignment

In Figure 1, we depict several cases in which the lack of proper alignment harms clustering. To be more explicit, we further conclude three propositions that support investigating the necessity of alignment, and a typical cluster-level alignment pitfall is discussed under simplified settings of multi-omics clustering problems in Supplementary Information. Inspired by recent contrastive learning algorithms, MoClust learns representations by maximizing agreement between different omics data from the same cell via a contrastive loss in the latent space.

For latent feature $z_{i}^{\left(u\right)}$ obtained from the encoder for the *u*-th omic with cell *i* as input. Assume that the predicted soft label of cell *i* is $\alpha_{i}$. Let latent features $z_{i}^{\left(u\right)}$ and $z_{j}^{\left(v\right)}$ be a positive pair if and only if they are representations of the same cell’s different omics data, which are i=j,u≠v. Then, the set of latent features that can be used to construct a negative pair with $z_{i}^{\left(u\right)}$ is

(5) $$N_{i}=\{z_{j}^{\left(v\right)}|j\neq i,\text{argmax}\alpha_{i}\neq \text{argmax}\alpha_{j}\}$$

which consists of all omics of all other cells that were assigned to a different cluster than that of cell i. Let $\text{sim}(u,v)=u^{⊤}v/||u||||v||$ denote the dot product between $\ell_{2}$ normalized ***u*** and ***v*** (i.e. cosine similarity). Compared to the popular Euclidean distance, cosine similarity focus on directions of the vectors rather than absolute values and thus alleviate the problem of inconsistent metrics across different omics. The loss function for a positive pair of latent features $\left(z_{i}^{\left(u\right)},z_{i}^{\left(v\right)}\right)$ is defined as

(6) $$\ell_{i,j}=-\text{log}\;\frac{\;\text{exp}\;\left(\text{sim}\left(z_{i}^{u},z_{i}^{v}\right)/\tau \right)}{\sum_{t=u,v}\sum_{z_{j}^{w}\in {\text{Nega}}_{i}}\;\text{exp}\;\left(\text{sim}\left(z_{i}^{t},z_{j}^{w}\right)/\tau \right)}$$

where ${\text{Nega}}_{i}$ is constructed by uniformly sampling a fixed number of latent features from $N_{i}$. This loss term ensures that we only repel representations of cells that were assigned to different clusters by the clustering module. The total contrastive loss is constructed by summing all $\ell_{i,j}$ as

(7) $$L_{\text{contrastive}\;}=\frac{1}{nM(M-1)}\sum_{i=1}^{n}\sum_{v=1}^{M}\sum_{u=1}^{M}I_{\left\{u\neq v\right\}}\ell_{i}^{\left(uv\right)}$$

The contrastive alignment is thus at the sample level. To reduce annoying tuning of parameters, we multiply the contrastive loss by the weight of the least informative omics so that the strength of the contrastive alignment can be automatically adjusted.

### 2.3 Multi-omics clustering

To obtain the final cluster assignments, we add a fully connected layer after the fusion layer, producing *h_i_*, the hidden representation for cell *i*. Another fully connected layer with a softmax activation is followed to obtain the *k*-dimensional soft label *α_a_*.

As is admitted in most deep learning clustering methods, we assume that the denoising autoencoder is capable of obtaining a low-dimensional representation which captures most of biological information in the input omics data. Basing on this assumption, we designed three clustering losses to find a better space where the clusters are compact and separable, thus making it easy to distinguish among different clusters. Additionally, we also want the cluster assignments for cells to be deterministic and the assignment vectors for clusters to be orthogonal. Inspired by former works on divergence-based clustering (Kampffmeyer *et al.*, 2019), we designed a Deep Divergence-based Clustering (DDC) loss based on CS divergence to conduct clustering over *z_i_*. DDC loss consists of three parts, ensuring the separability and compactness of clusters, closeness of cluster assignments to simplex corners and orthogonality of cluster assignments, respectively.

Considering k≥2 distinct probability density functions (PDFs), CS divergence is defined as

(8) $$D_{\text{cs}}\left(p_{1},\ldots ,p_{k}\right)=-\text{log}\;\left(\frac{1}{k}\sum_{i=1}^{k-1}\sum_{j>i}\frac{\int p_{i}(x)p_{j}(x)dx}{\sqrt{\int p_{i}^{2}(x)dx\int p_{j}^{2}(x)dx}}\right)$$

For a pair of PDFs, *p_i_* and *p_j_*, we have $0\leq D_{\text{cs}}\left(p_{1},p_{2}\right)<\infty$, where we obtain the minimum value iff *p_i_* = *p_j_*. Assume that we have a *n *×* k* assignment matrix $A=[\alpha_{a,i}]$. For the first term of clustering loss, we make use of the divergence in Equation (8) to measure the distance between clusters. Since the underlying true densities at point *x*, $p_{i}(x)$ is unknown, we replace them with soft cluster assignments of cluster *i* for cell as $a\;\alpha_{a,i}$ and configure the optimization object with a Gaussian kernel having bandwidth *σ*. To be more explicit,

(9) $$L_{1}=-D_{\text{cs}}\left(\alpha_{1},\ldots ,\alpha_{k}\right)=\frac{1}{k}\sum_{i=1}^{k-1}\sum_{j>i}\frac{\alpha_{i}^{T}K\alpha_{j}}{\sqrt{\alpha_{i}^{T}K\alpha_{i}\alpha_{j}^{T}K\alpha_{j}}}$$

where $\alpha_{i}$ is the *i*-th column of **A**, the similarity values in **K** follows $\kappa_{ij}=\text{exp}\;(-||h_{i}-\;h_{j}|{|}^{2}/\left(2\sigma^{2}\right))$, and *σ* is a hyperparameter. Minimizing $\ell_{1}$ pushes cosine similarity between cells in the same cluster to be small and the similarity between cells in different clusters to be large, making clusters separable and compact.

For the second term of clustering loss, we make use of the divergence in Equation (8) to measure the distance between soft cluster assignment vectors for cells and simplex corners. Suppose that $\alpha_{a}$ is the *a*-th row of **A**, and $e_{j}$ is corner *j* of the standard simplex in $R^{k}$. We define an additional term for the loss function $m_{a,j}=\text{exp}(-||\alpha_{a}-e_{j}||)$, and $m=[m_{a,j}]$

(10) $$L_{2}=-D_{\text{cs}}\left(m_{1},\ldots ,m_{k}\right)=\frac{1}{k}\sum_{i=1}^{k-1}\sum_{j>i}\frac{m_{i}^{T}Km_{j}}{\sqrt{m_{i}^{T}Km_{i}m_{j}^{T}Km_{j}}}$$

where $m_{i}$ is in the *i*-th column of **m**. Minimizing this part of loss encourages the cluster assignment vectors to be close to the standard simplex in $R^{k}$.

The third part is designed as the strictly upper triangular elements of $A^{T}A$, where **A** is the *n *×* K* soft assignment matrix,

(11) $$L_{3}=\text{triu}(A^{T}A)$$

where triu(*X*) refers to the strictly upper triangular elements of matrix *X*. The DDC module consists of inner products between cluster assignment vectors. Cluster assignment vectors are orthogonal if and only if these inner products are zero. Optimizing $L_{3}$ encourages the cluster assignment vectors for different objects to be orthogonal.

The final clustering loss is the sum of these three terms:

(12) $$L_{\text{cluster}\;}=L_{1}+L_{2}+L_{3}$$

Finally, the total loss we use to train MoClust is

(13) $$L=L_{\text{ZINB}}+L_{\text{cluster}\;}+\delta \cdot \min\left\{w_{1},\ldots ,w_{M}\right\}L_{\text{contrastive}}$$

where $L_{\text{cluster}\;}$ is the clustering loss we defined in Eq. (12), and *δ* is a hyperparameter which influences the strength of the contrastive loss. $w_{1},\ldots w_{M}$ are the fusion weights defined in Equation (4).

### 2.4 Automatic doublet discovery

MoClust can also find doublets efficiently and effectively without manually setting a threshold. Before identifying the doublets, a pretraining process is needed. The pretraining loss to be optimized is:

(14) $$L_{\text{pre}}=\gamma *L_{\text{ZINB}}+L_{1}+\delta \cdot \min\left\{w_{1},\ldots ,w_{M}\right\}L_{\text{contrastive}\;}$$

Formally, for each cluster *i* we define a prototype

(15) $$P_{i}=\frac{1}{\left|S_{i}\right|}\sum_{z_{a}^{\left(r\right)}\in S_{i}}z_{a}^{\left(r\right)}$$

where $z_{a}^{\left(r\right)}$ refers to the latent feature of transcriptomic data of cell *a*. *S_i_* consists of all $z_{a}^{\left(r\right)}$ that cell *a* is assigned to cluster *i* by MoClust. Given a noise level *ϵ*, we randomly sample *n_i_* cells from Gaussian distribution $N(P_{i},\epsilon )$ to construct a pseudo samples set $P_{i}$. Total positive samples set $\text{Posi}=\overset{k}{\underset{i=1}{\cup }}P_{i}$. For arbitrary pseudo samples $\hat{z_{ia}},\;\hat{z_{jb}}$ from different sets $P_{i}$ and $P_{j}$, we define their linear fusion as pseudo doublet:

(16) $$\hat{z_{\lambda }}=\lambda \hat{z_{ia}}+(1-\lambda )\hat{z_{jb}}$$

where *λ* is a parameter to control the fusion weights and we fixed λ=0.5 in all experiments. We sample $N_{n}$ pairs to compute $\hat{z_{\lambda }}$. MoClust predicts their soft labels $\hat{\alpha_{1}},\hat{\alpha_{2}},\ldots ,\hat{\alpha_{N_{n}}}$ and computes their entropy:

$$\hat{\epsilon_{a}}=\sum_{i=1}^{k}\hat{\alpha_{ai}}\;\text{log}\;\hat{\alpha_{ai}}$$

Given a level *α*, we compute the *α*-quantile from experimental distribution acquired from $\hat{\epsilon_{1}},\hat{\epsilon_{2}},\ldots ,\hat{\epsilon_{N_{n}}}$. We mark a cell as ‘Doublet’ if the entropy of its prediction is larger than $F_{\alpha }$.

## 3 Results

### 3.1 Moclust integrates transcriptome-proteome data

MoClust is suitable for clustering single-cell RNA-seq and antibody-derived tags data. To validate the effectiveness and efficiency of MoClust clustering CITE-seq data, we selected four real-world CITE-seq datasets, 10X10k, 10XInHouse, Spleen and Lymph datasets. The details of each dataset are listed in Supplementary Information. A series of simulation datasets were also generated by R package Splatter to verify the performance of MoClust under various circumstances, some with low-quality protein data and some with a large variety of proteins. The detailed settings of simulation datasets are listed in Supplementary Table S1.

We applied several state-of-the-art single-cell multi-omics clustering methods available for transcriptomic and proteomic data over these for datasets for comparison, namely, Seurat V4, CiteFuse (Kim *et al.*, 2020), jointDIMMSC (Sun *et al.*, 2018), BREMSC (Wang *et al.*, 2020), scMM (Minoura *et al.*, 2021), scCTClust and TotalVI. We also used integrative methods Liger, Harmony and UnionCom to obtain low-dimensional representations and apply k-means algorithm to get clustering results for comparison. The effectiveness of each method was evaluated by normalized mutual information (NMI) and adjusted Rand index (ARI) which are broadly used in clustering analysis.

**Fig. 3.**
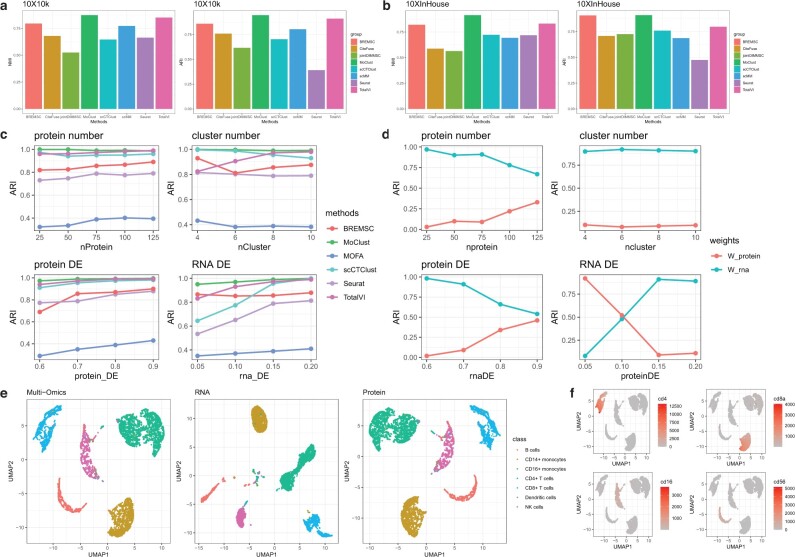
MoClust integrates scRNA and protein data. (**a** and **b**) Performance of MoClust and competing methods by NMI and ARI over real CITE-seq datasets 10X10k and 10XInhouse. (**c**) CITE-seq simulation experiments. All simulated data were generated by Splatter, and the performance of each method was evaluated by ARI. (**d**) The change of fusion weights learned by MoClust when applying on different simulated datasets. (**e**) Two-dimensional visualization of latent features extracted by MoClust over the 10X10k dataset by the UMAP dimension reduction method. From left to right, fused features, RNA features and protein features are listed and colored by true cell types. (**f**) UMAP visualization of the fused feature applying MoClust over 10X10k dataset, colored by the expression of different marker proteins

Casting our sight on Figure 3a and b, we can see that MoClust outperforms all other competing methods over 10X10k and 10XInHouse datasets. TotalVI gains better NMI and ARI than Seurat and CiteFuse, indicating the advantage of a deep learning algorithm in characterizing the feature of high-dimensional data. BREMSC behaves pretty well in these two datasets, especially over the 10XInHouse dataset, only slightly worse than our MoClust. However, utilizing a vanilla Markov Chain Monte Carlo (MCMC), BREMSC can hardly be praised as scalable, taking hours over the 10X10k dataset with 7865 cells. We use the two dimensional visualization method Uniform Manifold Approximation and Projection (UMAP) to investigate the latent structure of MoClust. In Figure 3e, we show fused representation, RNA and protein low-dimensional representations of MoClust applied on the 10X10k dataset, colored with the true cell types. We can see that the protein data are evidently more informative clustering than RNA data, presenting more compact and separable clusters. With the powerful contrastive alignment module, MoClust prioritizes omics by assigning the fusion weight for RNA as 0.282 and weight for protein as 0.718, correctly identifying the protein omics as more informative. Thus MoClust enriches cluster-specific information and obtains a representation more suitable for clustering than using single omics.

To investigate whether MoClust is capable of catching the relationship between markers and cell types, we colored the visualization of fused features by the antibody-derived tags (ADT) counts according to marker protein. For instance, in Figure 3f, we see a high correlation between the representation of marker protein CD56 and B cells. Other similar results also validate the utility of interpretable latent representations learned by MoClust. Supplementary Figure S2 displays the latent visualization of several competing methods. CiteFuse separate CD4+ T cells into several subgroups. Seurat gets dispersed clusters for CD4+ T cells and CD8+ T cells, while TotalVI finds it hard to distinguish CD4+ T cells from CD8+ T cells. Without distribution alignment, representation for the more informative omics gets perturbed after fusing, leading to less compact and separable clusters, which hampers clustering. We also displayed the UMAP plot of the fused representation of a simplified implementation of MoClust which is trained without the contrastive module. Compared to the UMAP for protein representations in Figure 3e, we can easily see that B cells, NK cells and CD14 monocytes get closer to each other and that some CD4+ T cells are mixed together with NK cells. This offers another example showing that linear combination without alignment does not benefit clustering analysis. Additionally, we drew the dotplot of known marker proteins against predicted clusters or true cell types in Supplementary Figure S1. In this way, MoClust may help researchers find undiscovered relationship between proteins and cell types.

We also applied MoClust and competing methods over a series of simulated datasets. By varying the differential expression probability of RNA and protein, we simulated multi-omics datasets of different RNA or protein quality levels. We also generated datasets with different protein numbers and cluster numbers to simulate different circumstances of sequencing. The ARI and NMI of MoClust and competing methods are shown as line charts in Figure 3c and Supplementary Figure S1. In general, MoClust has substantial advantages over different settings of parameters, especially on datasets with poor quality of protein or RNA data. To further explore whether the fusion weights can be adaptively learned by MoClust, we display the change of fusion weights while varying the number of protein, clusters and differential expression probability of RNA or protein in Figure 3d. We can clearly see that the protein weight increased along with the dimension of protein data, as well as the differential expression probability of protein. RNA weights also got larger when the differential expression probability went up. Both weights are not sensitive to the number of clusters. It indicates that MoClust can well prioritize different omics by their contributions to clustering.

To investigate whether MoClust is scalable, we used R package Splatter to simulate a series of CITE-seq datasets with different number of cell samples and features and record the running time of different methods. The RNA DE probability is fixed as 0.15, protein DE probability as 0.7, number of clusters as 10. As is shown in Supplementary Figure S1f, deep learning-based methods MoClust, scCTClust and TotalVI are all of good scalability and not very sensitive to the dimension of features. Seurat and CiteFuse are also not sensitive to the number of features as they selected highly variable genes during data preprocess. But CiteFuse is extremely sensitive to the number of samples with a computational complexity of O$\left(n^{3}\right)$. MCMC-based methods jointDIMMSC and BREMSC also cost more time clustering CITE-seq dataset, the gap between the running times of BREMSC and MoClust becomes larger as the number of samples or features increases.

### 3.2 MoClust integrates transcriptome-epigenome data

MoClust can also be applied on scRNA-seq and scEpigenome data for integrative analysis. We examined MoClust on three published datasets, including SNARE-seq datasets CellLine and 10XPBMC, as well as SHARE-seq (Clyde, 2020) dataset Ma-2020. The detailed information of these datasets is listed in the Supplementary Information. For comparison, we also applied several state-of-the-art clustering methods for scRNA-seq and scATAC-seq data, including Seurat V4, MOFA+, scAI, cobolt, scMVAE, DCCA and MultiVI. Integrative methods Liger, Harmony and UnionCom (Cao *et al.*, 2020) were also applied on these datasets to obtain joint embeddings on which kmeans++ is utilized to get a clustering results. Detailed settings for competing methods can be found in Supplementary Information.

**Fig. 4.**
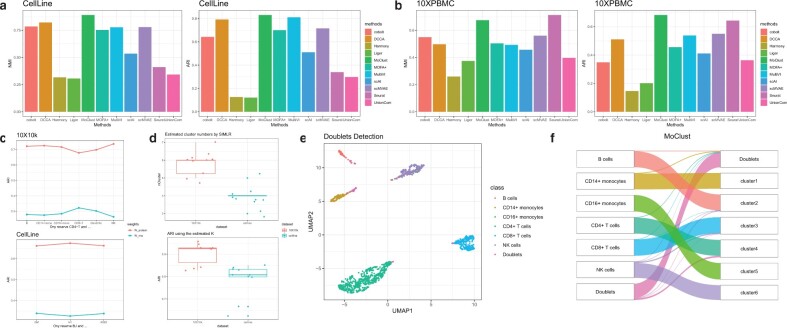
(**a** and **b**) Performance of MoClust and competing methods by NMI and ARI over the real RNA+ATAC multi-omics datasets CellLine and 10XPBMC. (**c**) The change of fusion weights when clustering on different subgroups of cell types. (**d**) The estimated numbers of clusters by SIMLR with 10 different random seeds. And the ARI of MoClust using the estimated number of clusters. (**e** and **f**) UMAP visualization and Sankey plot of the clustering results performed by MoClust on the 10XInHouse dataset. Doublet detection module is employed in according experiments

In Figure 4a and b, we display the NMI and ARI of each methods clustering CellLine and Ma-2020 single-cell multi-omics data. First focusing on the CellLine dataset results, not surprisingly, MoClust gained the highest NMI and ARI over all eleven methods. DCCA follows MoClust, performing well on CellLine dataset. However, tuning the hyperparameters of DCCA, especially tuning the loops for cross-omics cycle attention, is quite time-consuming. Other Deep generative methods cobolt, scMVAE and MultiVI also deliver rather satisfactory results, but still not as good as MoClust. Matrix factorization based methods MOFA+ and scAI did not present a significant performance, ranking median among all methods in NMI and ARI. Seurat performed poorly with an NMI around 30%. Integrative methods Liger, Harmony and UnionCom also got unsatisfactory results which may be imputed on the separateness of dimension reduction and clustering. And cobolt seemed to be collapsed applying on CellLine data under default parameters. To better demonstrate the powerful alignment ability of MoClust, we visualized the latent features of RNA data, ATAC data and fused data with UMAP over the CellLine dataset. We can find in Supplementary Figure S2d that there are significant difference between the latent features of RNA and ATAC data, indicating that an effective alignment method is urgently needed. With the powerful contrastive learning-based alignment method, MoClust successfully identified that the contribution of RNA-seq is bigger than that of ATAC-seq, assigning the fusion weight of two omics at 0.561 and 0.439, separately. The alignment module also guides the dimension reduction of two omics, helping MoClust find a latent space where clusters are separable and compact, thus gaining high NMI and ARI. UMAP visualizations for competing methods are listed in Supplementary Information.

10XPBMC is a published SNARE-seq dataset that can be downloaded from 10X Genetics website. Among all methods, MoClust got the highest ARI applying on 10XPBMC dataset and a NMI only slightly lower than Seurat. As we utilized the Seurat annotations based on transcriptome state as approximate truth, the tiny gap between NMIs of MoClust and Seurat is unsurprising. We also performed all methods over the large-scale SHARE-seq dataset Ma-2020, the detailed results of NMI and ARI can be seen in Supplementary Figure S2a. MoClust maintained its dominant role on Ma-2020. MultiVI also gained high NMI and ARI, slightly lower than MoClust. However, the running time of MultiVI exploded as the number of ATAC peaks grows rapidly, taking tens of hours to learn representations from Ma-2020 dataset with over 300k peaks. In conclusion, MoClust outperformed several state-of-the-art methods integrating scRNA and scATAC data of different scales and constitution.

### 3.3 MoClust detects doublets effectively

MoClust implemented an automatic doublet detection module to identify doublets without manually setting a threshold. First, we pretrain MoClust by optimizing $L_{\text{pre}}$ and then generate pseudo doublets by linearly fusing representative cells of different clusters. Given a confidence level *α*, the *α*-quantile of the empirical distribution obtained from the pseudo counts will serve as a threshold for identifying real doublets. We applied our method on a perturbed 10XInHouse CITE-seq dataset. We randomly chose 80 pairs of cells, each pair from distinct cell types, and averaged the RNA and ADT counts of cells from the same pair to simulate doublets. Figure 4e displayed the UMAP visualization of the fused features generated by MoClust applied to the 10XInHouse perturbed dataset. We can clearly see that doublets are mapped far away from cluster centers, allowing us separate them from normal samples. Figure 4f shows the Sankey plot between true cell types and clusters predicted by MoClust. Sixty-eight out of a total 80 doublets were successfully identified by MoClust, and only 30 normal samples were mistaken for doublets during the experiment.

### 3.4 Ablation study

We also verified stability, while varying four hyperparameters introduced in our model, including *σ*, *τ*, *δ* and *γ*, which can be seen in Supplementary Figure S2e. We fix the training dataset and CITE-seq dataset 10X10k and tuned those hyperparameters to observe any change in MoClust’s performance. We found the performance of MoClust to be insensitive to *τ*, *δ* and *γ*. We also found that the adequate intervals for *σ* were similar across different experiments. Optimal hyperparameters for MoClust through all experiments are listed in details in Supplementary Table S2. We further gave the recommended hyperparameters for clustering CITE-seq and SNARE-seq data using MoClust. The clustering performance of MoClust over real datasets using recommended settings are also listed in Supplementary Table S2.

To investigate the importance of distribution alignment, we trained MoClust without the contrastive loss on CITE-seq datasets 10X10k and 10XInhouse. For 10X10k, without the contrastive module, the ARI deteriorated from 94.5% to 85.9% while the NMI decreased from 89.2% to 83.1%. In Figure 3e and Supplementary Figure S2a, we displayed the UMAP visualizations of fusion representations applying MoClust with and without contrastive module on the 10X10k dataset. Without contrastive distribution alignment, CD4+ T cells and CD8+ T cells become more disperse, while NK cells, B cells and CD14+ monocytes become harder to distinguish. Similar results were obtained over the 10XInHouse dataset.

To investigate whether the fusion weights is strongly correlated with cell types or they are mainly related to the quality of omics, we selected different subgroups of cell types appeared in 10X10k dataset and applied MoClust on cells from selected cell types. In Figure 4c, we can see that the fusion weights did not vary violently on different subgroups of cell types. Similar results were obtained using SNARE-seq dataset CellLine. It indicates that MoClust can learn fusion weights as the contributions of omics made to clustering.

Through all experiments, we trained each model with the number of clusters the same as the number of different cell types appeared in the dataset. In practice, when the true number of cell types can hardly obtain, we suggest using scRNA-seq analyzing tool SIMLR (Wang *et al.*, 2017) to estimate the number of clusters. In Figure 4d, we estimated the number of clusters of datasets 10X10k and CellLine by performing SIMLR on the transcriptome data. All experiments were repeated with ten different random seeds, the estimated number of clusters and the ARIs of MoClust with the estimates are shown in boxplot. We can see the gap between the estimates and truth is small while MoClust can still perform well when the cluster number is slightly biased.

We also evaluated the contributions three parts $L_{1},L_{2}$ and $L_{3}$ of the clustering loss $L_{\text{cluster}}$ made to the final clustering performance. We trained MoClust on 10X10k dataset. Taking out $L_{1}$, MoClust got an ARI of 67.6% and a NMI of 64.7%. Setting the clustering loss as $L_{1}+L_{3}$, ARI and NMI became 66.9% and 66.3%, while setting the clustering loss as $L_{2}+L_{3}$ ARI and NMI became 88.0% and 85.7%.

### 3.5 Grid search for deep learning methods

To further validate that MoClust performs better than existing deep learning methods when clustering single-cell multi-omics data, we selected two other benchmark datasets, namely bone marrow mononuclear cell (BMMC)-cite and BMMC-multiome, with known ground truth labels to conduct grid search for MoClust and all competing deep learning methods. The detailed information of datasets and the criterion for hyperparameters selection are described in the Supplementary Information. We tuned the selected hyperparameters of MoClust and other methods over these two datasets and evaluated their performance by NMI. The results are shown in the form of heatmaps in Supplementary Figure S3. The best performance of MoClust surpassed the best of all other deep learning methods.

## 4 Discussion

MoClust offers a new perspective of integrative analysis for single-cell transcriptome, epigenome and proteome. Clustering methods for single-cell data have long been obsessed over identifying rare cell types. In 10XInHouse dataset, MoClust also found it hard to distinguish rare cell type ‘CD16 + monocytes’ from ‘CD14 + monocytes’ and we are still making efforts to make MoClust capable of identifying these rare types. To avoid collapse of small clusters, i.e. rare cell types, we added a selective $L_{\text{kl}\_\text{div}}$ loss, which is explained detailed in Supplementary Information, to the optimization term of the clustering module. We chose the CITE-seq dataset Total_Spleen for further experiments, as it contains over 4000 different cells from 21 cell types, several having 50 or fewer samples. We focused on rare cell types Ifit3-high CD4 T and Ifit3-high CD8 T cells, containing 30 and 25 samples, respectively. Trained with $L_{\text{kl}\_\text{div}}$ loss, only 3 of 30 Ifit3-high CD4 T cells were clustered together with CD4 T cells, while 19 of 25 Ifit3-high CD8 T samples were successfully separated from CD8 T cells. However, the accuracy for big clusters decreased at the same time, indicating that we have to find a balance between clustering accuracy and the ability to distinguish rare types. In the future, we foresee further extending our model to reduce the proportion of mis-clustered cells under all situations.

## 5 Conclusion

When applied to CITE-seq and SNARE-seq data datasets, MoClust outperforms other state-of-the-art methods for scRNA and protein joint clustering analysis. MoClust shows remarkable NMI, ARI over four different datasets and presents more separable and compact clusters than any other methods. MoClust successfully captured most biological biases and correctly distinguish cells from different cell types in most cases,

In practice, single-cell clustering methods are often confused by doublets which typically arise owing to errors in cell sorting or capture. MoClust is armed with an automatic doublet detection module. With experiments over the 10XInHouse dataset, the efficiency and effectiveness of MoClust in detecting doublets and enhancing its clustering performance can be clearly seen. We believe that the deliberately designed representation alignment afford MoClust more practicality and power when compared to any other state-of-the-art methods now available.

## Supplementary Material

btac736_Supplementary_Data

## Data Availability

The data underlying this article are available in the article and in its online supplementary material.
