# Supplementary material for: Clustering single-cell multi-omics data with MoClust
Source: Bioinformatics. 2022 Nov 16;39(1):btac736. doi: 10.1093/bioinformatics/btac736 (PMC9805570; doi:10.1093/bioinformatics/btac736)
Supplement: btac736_Supplementary_Data [file btac736_supplementary_data.pdf]

Supplementary meterials for
Clustering single-cell multi-omics data with MoClust

Musu Yuan, Liang Chen and Minghua Deng

November 2, 2022

Appendix

The necessity and pitfalls of distribution alignment in multiomics clustering

First, we consider an simplified version of multiomics clustering problem. This allow us to investigate what will happen when conducting clustering algorithms over multiomics data, without proper distribution alignment techniques:

Proposition 1. Suppose our dataset consists of two omics and k ground truth cell types. And we wish to cluster the data according to this ground truth clu stering. For the first omic, all observations from the same ground truth cell type are located at the same point in the input space, formally $x_{11}, x_{12}, ..., x_{1k}$. And for the second omic, all observations are located at the same point x_2, indicating low quality omic data. We utilize the sum of $\mathcal{L}_2$ distance between cluster centers to evaluate the separableness of clusters (the larger the better). The $\mathcal{L}_2$ sum is $\sum_{i \neq j} ||x_{1i} - x_{1j}||_2$ for the first omic and 0 for the second omic. Without distribution alignment, we simply averaged the representations of two omics and the $\mathcal{L}_2$ sum becomes:

$$\sum_{i \neq j} ||\frac{1}{2}(x_{1i} + x_2) - \frac{1}{2}(x_{1j} + x_2)||_2 = \frac{1}{2} \sum_{i \neq j} ||x_{1i} - x_{1j}||_2$$

which means multiomics clustering would get worse clustering performance than single-omic clustering over the first omic, as the clusters are less separable.

Proposition 2. Suppose our dataset consists of two omics and k ground truth cell types. And there are m samples from cell type i. For the first omic, all observations from the same ground truth cell type are located at the same point in the input space, formally $x_{11}, x_{12}, ..., x_{1k}$. And for the second omic, observations from cell type i are assigned into two different clusters. m_1 of all m samples are located at point x_{2i}^1, the rest m_2 samples are located at point x_{2i}^2. We adopt the sum of $\mathcal{L}_2$ distance between samples from the same ground truth cell type to evaluate the compactness of clusters (the smaller the better). The $\mathcal{L}_2$ sum is 0 for the first omic and $m_1 m_2 ||x_{2i}^1 - x_{2i}^2||_2$ for the second omic.Without distribution alignment, we simply averaged the representations of two omics and the $\mathcal{L}_2$ sum becomes:

$$m_1 m_2 ||\frac{1}{2}(x_{1i} + x_{2i}^1) - \frac{1}{2}(x_{1i} + x_{2i}^2)||_2 = \frac{1}{2} \sum_{i \neq j} ||x_{2i}^1 - x_{2i}^2||_2$$

which is larger than the $\mathcal{L}_2$ sum of the first omic, indicating less compact clusters than single omic circumstance.

Even when data from all omics are perfectly aligned, the performance of multiomics clustering method cannot be guaranteed. Here we consider an idealized version of multiomics clustering problems to show several pitfalls existing alignment methods may fall into.

Proposition 3. Suppose our dataset consists of O omics and k ground truth cell types. For each view, all observations that belong to the same ground truth cluster, are located at the same point in the input space. For a given omic o, $o \in 1, 2, ..., O$, the number of clusters in the input space is k_o. The omics are mapped to representations using omic-specific encoders, and subsequently fused according to a linear combination with unique weights. Then, if the distributions of representations from different omics are perfectly aligned, the maximum number of unique clusters after fusion is:

$$k_{\text{fused}}^{\text{aligned}} = \min\{k, (\min_{o=1,2,...O} k_o)^O\}$$

and if they are not aligned.:

$$k_{\text{fused}}^{\text{not\_aligned}} = \min\{k, \prod_{o=1}^{O} k_o\}$$

In extreme cases, when there is a i satisfying $k_i = 1$ which means this omic contains no useful information for clustering, $k_{\text{fused}}^{\text{aligned}} = k_i^O = 1$. Alignment thus prevents the suppression of this omic, and makes it harder to discriminate between clusters in the representation space. A stronger assumption about $k_1, k_2, ...k_O$ is that $k_1 = k_2 = ... = k_O = k$, supposing that we are able to discriminate between all clusters in all omics. This assumption is adopt by many end-to-end alignment methods, such as DCCA. In this case $k_{\text{fused}}^{\text{aligned}} = k_{\text{fused}}^{\text{not\_aligned}} = k$, both alignment-based models and non-alignment-based models are possible to perfectly cluster the data. Unfortunately, this assumption is too strong for most circumstances since the quality of different omics data is usually of great diversity.

Data Availability

In section 3.1 and 3.2, we validated the performance of MoClust clustering several single-cell multi-omics datasets. These datasets consist of four CITE-seq datasets, two SNARE-seq datasets and one SHARE-seq dataset. The detailed information of these datasets is listed as follows.

10X10k PBMC dataset is a published human PBMC CITE-Seq dataset downloaded from 10X Genomics website (https://support.10xgenomics.com/single-cell-gene-expression/datasets/3.0.0/pbmc 10k protein v3). A total of 7,865 cells from a healthy donor were stained with 14 TotalSeq-B antibodies, including CD3, CD4, CD8a, CD14, CD15, CD16, CD19, CD25, CD45RA, CD45RO, CD56, CD127, PD-1 and TIGIT. Cell-matched scRNA-Seq data are available. The cell type of each sample is identified based on the biological knowledge of both protein and gene markers which is illustrated in Figure S1 in [7] and is set as the approximate truth through our experiments.

10XInHouse PBMC dataset is a in-house CITE-seq dataset of human PBMC from a healthy donor under IRB approval from the University of Pittsburgh, generated by [7]. 1372 cells were stained with Totalseq-A from BioLegend and are prepared using the 10x Genomics platform with Gel Bead Kit V2. The prepared assay is subsequently sequenced on an Illumina Hiseq with a depth of 50K reads per cell. Cells in this dataset are measured for their surface marker abundance through CITE-seq. Ten surface markers are measured for every cell: CD3, CD4, CD8a, CD11c, CD14, CD16, CD19, CD56, CD127 and CD154. Cell Ranger 3.0 was used to process the data and generate UMI matrix for the downstream analysis. The cell type is annotated by [7], based on the biological knowledge of both protein and gene markers.

Spleen & Lymph nodes datasets are separated from the SLN111-D1 dataset generated by [3]. The SLN111-D1 dataset is obtained by conducting CITE-seq experiment over cells were from two wild-type mice. In the CITE-seq experiment run, cells were stained with a panel of barcoded antibodies, containing 111 different antibodies. The cell types of each dataset are identified by TotalVI based on the biological knowledge of both protein and gene markers and are set as the approximate truth through experiments.

10XPBMC dataset is a published SNARE-seq dataset of human PBMC which can be downloaded from 10X Genomics website (https://support.10xgenomics.com/single-cell-multiome-atac-gex/datasets/1.0.0/pbmc_granulocyte_sorted_10k). A total of 11909 cells were stained with SNARE-seq and is divided into 20 different cell types by [4] based on transcriptome state.

Cell Line dataset with 1047 cells with paired scRNA-seq and scATAC-seq data is drawn from mixtures of cultured human BJ, H1, K562 and GM12878 cells lines by SNARE-seq ([2]).

Ma-2020 dataset containing 34 774 cells with paired scRNA-seq and scATAC-seq data is derived from adult mouse skin by SHARE-seq ([6]). All cells are annotated by [6] based on both transcriptome and epigenome states. The cell types obtained are set as approximate truth through our experiments.

BMMC datasets. To better demonstrate the performance of MoClust using recommended parameters, we used the benchmark dataset which was provided in the course of the NeurIPS 2021 competition and for which the ground-truth cell identity labels are known ([5]). This dataset was the first available multi-omics benchmarking dataset for single-cell biology. It mimics realistic challenges researchers are faced with when integrating single-cell multi-omics data, e.g., by incorporating nested donor and site batch effects. Specifically, the NeurIPS benchmark dataset is a multi-donor (10 donors), multi-site (4 sites), multi-omics human bone marrow dataset comprising two data types sequenced by 10X Genomics ([1]):

- BMMC-cite: CITE-seq data with 81,241 cells, where for each cell RNA gene expression (GEX) and cell surface protein markers using antibody-derived tags (ADT) are jointly captured.
- BMMC-multiome: 10X Multiome assay data with 62,501 cells, where nucleus GEX and chromatin accessibility measured by assay for transposase-accessible chromatin (ATAC) are jointly captured.

In total, this dataset contained information on the accessibility of 119,254 genomic regions, the expression of 15,189 genes, and the abundance of 134 surface proteins. The dataset can be accessed via https://www.ncbi.nlm.nih.gov/geo/query/acc.cgi?acc=GSE194122.

Competing methods

For all methods, we set the number of clusters same as the number of true different cell types across all experiments.

CITE-seq Experiments. For BREMSC and joint-DIMMSC, we followed the examples in their project homepage (https://github.com/tarot0410/BREMSC), utilizing function "jointDIMMSC" with default parameters and function "BREMSC" with nChains=2, nMCMC=100 in all experiments.

For CiteFuse, we followed the vignette offered by the authors (https://sydneybiox.github.io/CiteFuse/articles/CiteFuse.html). We replaced K with the number of different cell types.

For scCTClust, we use the default parameters as is provided in the project homepage (https://github.com/ddb-qiwang/scCTClust-torch). For 10X10k, Spleen and Lymph datasets, the dimensions of RNA encoder are set as [256,64,32]. For 10XInHouse, the dimensions of RNA encoder are set as [32]. Hyper-parameter δ is fixed as 0.1 across experiments, while γ fixed as 1.0.

For scMM, we followed the tutorial given by authors . (http://htmlpreview.github.io/?https://github.com/kodaim1115/test/blob/master/tutorial.html). The batch size is tuned as 256, while the number of epochs is set as 100. For 10X10k and 10XInhouse datasets, the r_hidden_dim is fixed as 256, while the p_hidden_dim is fixed as 10. For spleen and Lymph datasets, the r_hidden_dim is fixed as 256, while the p_hidden_dim is fixed as 32.

For Seurat, we adopt the V4 version of Seurat and conducted clustering following (https://satijalab.org/seurat/articles/weighted_nearest_neighbor_analysis.html). We tuned the resolution for FindClusters to make sure that the cluster number equalling to the number of different cell types.

For TotalVI, we followed the tutorial offered in the homepage of scvi-tools (https://docs.scvi-tools.org/en/stable/tutorials/notebooks/totalVI.html). We set the training epochs as 300 during training. We also tuned the resolution in the leiden algorithm to ensure that the cluster number equalling to the number of different cell types.

SNARE-seq Experiments. For cobolt, we followed the tutorial (https://github.com/epurdom/cobolt/blob/master/docs/tutorial.ipynb). For CellLine dataset, the number of latent units is set as 10 while the maximal training epochs is set as 20. For 10XPBMC and Ma-2020 dataset, the number of latent units is set as 32 while the maximal training epochs is set as 100. We also tuned the resolution in the leiden algorithm to ensure that the cluster number equalling to the number of different cell types.

For scMVAE, we kept the hyperparameters as default settings given in the projects' homepage (https://github.com/cmzuo11/scMVAE). As the author has applied scMVAE on the CellLine dataset in original paper, we repeated the experiment under the same settings. For 10XPBMC and Ma-2020 dataset, we ran scMVAE_POE with the dimensions of encoder1 and encoder2 fixed as [1024,256,128] while Z_dim fixed ad the number of different cell types. The batch size is set as 256 in these two experiments.

For DCCA, we followed the tutorial (https://github.com/cmzuo11/DCCA/wiki/Analysis-of-cellMix-dataset-from-SNARE-seq-technology-by-DCCA-model). As the author has applied DCCA on the CellLine dataset in original paper, we repeated the experiment under the same settings. For 10XPBMC and Ma-2020 dataset, we ran DCCA with layer_e_2 fixed as [1024,256,128] while Zdim_1 and Zdim_2 fixed ad the number of different cell types. The batch size is set as 256 in these two experiments.

Since Liger, Harmony and UnionCom are single-cell multi-omics integration analysis tools rather than clustering tools, we conducted joint dimension reduction following their tutorials and obtained clustering results with kmeans++ algorithm under default settings. The tutorials of these three methods can be seen at (http://htmlpreview.github.io/?https://github.com/welch-lab/liger/blob/master/vignettes/online_iNMF_tutorial.html), (https://github.com/immunogenomics/harmony) and (https://github.com/caokai1073/UnionCom/blob/master/Examples/scGEM.ipynb). Over CellLine dataset, we pre-trained UnionCom for 3000 epochs and then trained UnionCom 200 epochs to embed the multi-omics representations. For Ma-2020, the numbers of pre-train epochs and train epochs are set as 5000, 500 to ensure convergence.

For MOFA+, we followed the vignette (https://raw.githack.com/bioFAM/MOFA2_tutorials/master/R_tutorials/SNARE_seq.html). The number of factors is set as the different cell types across all experiments to ensure that it is enough to distinguish all different cell types.

For Seurat, we followed the vignette (https://satijalab.org/seurat/articles/atacseq_integration_vignette.html). We tuned the resolution for FindClusters to make sure that the cluster number equalling to the number of different cell types.

For scAI, we did feature selection and run scAI, VscAI strictly as the examples provided in the project homepage did (https://github.com/sqjin/scAI). For the parameter nrun in function run_scAI, we set it as 2, 4, 10 when applying scAI over CellLine, 10XPBMC and Ma-2020 datasets to ensure convergence. K is tuned as the number of truly different cell types.

Additionally, the UMAP visualizations of each method across all datasets are obtained with the random seed fixed as 123 and the number of neighbors fixed as 20.

Identify rare cell types

We designed a selective loss function to avoid cluster collapse.

$$\mathcal{L}_{kl\_div} = D_{\mathrm{KL}}(a||t) = \sum_{i=1}^{K} a_i(\log a_i - \log 1/K)$$

where D_{KL} is the K-L divergence, a is the normalized frequency vector $(a_1, a_2, ...a_K)$, a_i is the proportion of cells predicted as cluster i in current iteration among all cells. t is the categorical distribution $(1/K, 1/K, ...1/K)$. K is the number of clusters.

Grid search for deep learning methods

To better demonstrate that MoClust is more powerful than existing deep learning single-cell multi-omics clustering methods, we conducted grid search for all deep learning methods simultaneously on benchmark datasets BMMC-cite and BMMC-multiome. First, we randomly subsampled 5000 cells from the origin datasets. Repeating it 10 times, we acquired 10 sub-datasets. Each method was applied on all 10 sub-dataset and the averaged NMI was computed.

As the number of hyper-parameters are not the same across these methods, also considering the cost of computational resources, we selected top 2 significant hyper-parameters to be tuned referring to their original papers for all methods. Other hyper-parameters were kept as default values through the whole experiment. We varied these top 2 significant hyper-parameters around their default values (setting them as median value if possible) as we believe these default values are well-tuned by researchers and can help the methods work well over similar single-cell multi-omics datasets. Details of what and why we selected is listed as follows.

MoClust: σ is the hyper-parameter that balance between significance of similar sample pairs and that of dissimilar sample pairs. When σ is small, the clustering loss concerns more about making dissimilar pairs with the same predicted cluster to be closer. The default value given by the author is 0.15 so we varied it between 0.10 and 0.25 in our experiment. δ is the hyper-parameter that controls the strength of contrastive module. The default value given by the author is 0.01 so we varied it between 0.1 and 0.001 in our experiment. We selected these two hyper-parameters since they affect the computation of clustering loss directly and MoClust is more sensitive to these two hyper-parameters rather than others such as 'latent dim' in practise.

scCTClust: σ is the hyper-parameter that balance between significance of similar sample pairs and that of dissimilar sample pairs. The default value given by the author is 0.15 so we varied it between 0.05 and 0.25 in our experiment. δ is the hyper-parameter that controls the strength of CCA loss. The default value given by the author is 0.001 so we varied it between 0.01 and 0.00005 in our experiment. In the original paper, authors conducted ablation study over these two hyper-parameters.

TotalVI: 'sample num' is the number of posterior samples used to get normalized gene and protein expressions. We selected it as significant hyper-parameter since the authors tuned it in their online tutorials, setting it as 25 when applying TotalVI on a real-world CITE-seq dataset. Thus we range the value of 'sample num' from 5 to 50. 'latent dim' is the dimension of its latent layer. The default value given by the author is 20 so we varied it between 10 and 50 in our experiment. We selected it as there are not many hyper-parameters needed to be tuned for TotalVI and the dimension of latent layer usually affect the performance of deep learning methods significantly in practise.

scMM: 'neighbor num' is the number of nearest neighbors used in the PhenoGraph clustering process and is selected since the author tuned it in tutorial. The default value given by the author is 20 so we varied it between 10 and 100 in our experiment. 'latent dim' is the dimension of its latent layer. The default value given by the author is 10 so we varied it between 5 and 100 in our experiment.

We selected it as there are not many hyper-parameters needed to be tuned for scMM and the dimension of latent layer usually affect the performance of deep learning methods significantly in practice.

Cobolt: 'max epoch' is the maximum number of training epochs. 'latent dim' is the dimension of its latent layer. The default value given by the author is 10 so we varied it between 5 and 100 in our experiment. The default value given by the author is 20 so we varied it between 10 and 100 in our experiment. We selected it as the author only tuned them and the learning rate in the online tutorial. We did not tune learning rate as it is tuned by the author over a similar SNARE-seq dataset and tuning the max epoch can also avoid the model to be underfitting or overfitting.

MultiVI: 'sample num' is the number of posterior samples used to get normalized gene expressions. The authors set it as 25 in their tutorial so we varied it between 5 and 50. We selected it as significant hyper-parameter since the authors tuned it in their online tutorials. 'latent dim' is the dimension of its latent layer. The default value given by the author is 20 so we varied it between 5 and 50 in our experiment. As MultiVI shares a similar framework with TotalVI, we selected similar hyper-parameters as we did for the latter.

scMVAE: 'networks' refers the three different learning strategies ('NN', 'POE', 'Direct') proposed in the original paper. They were compare in the paper and performed differently over various datasets. beta is the hyper-parameter appears in the ELBO term and is reported to influence the performance of VAE methods significantly by many researches. The default value given by the author is 1.0. We only varied it between 1.0 and 1.3 VAE gets the ability of disentanglement when beta is larger than 1.0.

DCCA: 'cycle num' refers to how many times the representations of different omics transfer from each other. The default value given by the author is 2 so we varied it between 1 and 4 in our experiment. It is especially mentioned by the author that DCCA is very sensitive to this parameter. beta is the hyper-parameter appears in the ELBO term and is reported to influence the performance of VAE methods significantly by many researches. The default value given by the author is 1.0. We only varied it between 1.0 and 1.3 VAE gets the ability of disentanglement when beta is larger than 1.0.

UnionCom: ρ is a training damping term that controls the strength of norm penalty. The default value given by the author is 10 so we varied it between 5 and 20 in our experiment. β is the trade-off parameter of structure preserving and point matching. The default value given by the author is 1 and is recommended to be larger so we varied it between 1 and 20 in our experiment. They are the only hyper-parameters used in the computation of the loss function which instantly influence the performance of the model despite from common hyper-parameters for training process such as learning rate.

In Figure S3, we displayed the results over BMMC-cite in the form of heatmaps, the warmer the colour of the block is, the higher NMI the method gained under according hyper-parameters setting. We can easily find in Figure S3(a) that only the performance of scMM is comparable with that of MoClust when both applied on CITE-seq dataset BMMC-cite. With default parameters, MoClust gained a NMI of 0.633, slightly worse that 0.638 which is acquired by scMM using optimal parameters.

Figure S3(b) depicted the performance of deep learning methods with various parameters settings applying on 10X multiome dataset BMMC-multiome. No method except scMVAE and DCCA are compatible with MoClust under the evaluation of NMI. Although the NMI gained by MoClust with default parameters is 0.519, lower than scMVAE's 0.542 and DCCA's 0.571 with optimal parameters, the best performance of MoClust, with a NMI of 0.581, is better than scMVAE and DCCA. We can also find that DCCA is extremely sensitive to the number of cycles, which is mentioned in the article.

Cell/Cluster	Cluster	Protein	Prob_{RNA}	$\text{Prob}_{\text{protein}}$
500	*	75	0.15	0.7
500	8	*	0.15	0.7
500	8	75	*	0.7
500	8	75	0.15	*

Table 1: Simulation data settings
For all experiments, each cluster contains 500 cells and we only vary one of the other four parameters, marked as '*' in the table. 'Cluster' and 'Protein' represent the number of clusters and proteins. Prob_{RNA} and $\text{Prob}_{\text{Protein}}$ indicate the differential expression probability of RNA and protein which are controlled by 'DE_prob' parameter of R package Splatter.

Dataset	Encoder1	Encoder2	γ_1	γ_2	σ	δ	NMI	ARI	r-NMI(r#)	r-ARI(r#)
10X10k	(256,64,32)	(32)	1.0	-	0.15	0.1	0.872	0.945	0.872(1)	0.945(1)
10XInHosue	(128,32)	(32)	1.0	-	0.15	0.1	0.914	0.909	0.914(1)	0.909(1)
Spleen	(256,64,32)	(32)	1.0	-	0.1	0.01	0.801	0.773	0.771(2)	0.759(2)
Lymph	(256,64,32)	(32)	1.0	-	0.1	0.01	0.672	0.670	0.659(3)	0.643(2)
CellLine	(128,32)	(128,32)	1.0	1.0	0.15	0.1	0.890	0.831	0.882(1)	0.821(1)
10XPBMC	(256,64,32)	(128,32)	1.0	0.8	0.15	0.1	0.674	0.682	0.674(2)	0.682(1)
Ma-2020	(256,64,32)	(128,32)	1.0	0.8	0.1	0.01	0.643	0.551	0.591(2)	0.527(1)

Table 2: Optimum hyper-parameters for MoClust.
"Encoder1" and "Encoder2" refer to the dimensions of layers after the input layer. γ_1 and γ_2 refer to the hyperparameters constrainting the strength of ZINB loss for transcriptome data and epigenome data. σ is used to compute the similarity matrix $\mathbf{K}$ in eq.(9). γ controls the strength of contrastive alignment. The optimal parameters for 10X10k and 10XPBMC are recommended settings for CITE-seq and SNARE-seq datasets. r-NMI(r#) represents the NMI of MoClust and its rank among all methods' NMI using recommended parameters settings. r-ARI(r#) is defined similarly.

Supplementary Figures

References

[1] Eva Brombacher, Maren Hackenberg, Clemens Kreutz, Harald Binder, and Martin Treppner. The performance of deep generative models for learning joint embeddings of single-cell multi-omics data. *bioRxiv*, 2022.

[2] Song Chen, Blue B Lake, and Kun Zhang. High-throughput sequencing of the transcriptome and chromatin accessibility in the same cell. *Nature biotechnology*, 37(12):1452–1457, 2019.

[3] Adam Gayoso, Zoë Steier, Romain Lopez, Jeffrey Regier, Kristopher L. Nazor, Aaron M. Streets, and Nir Yosef. Joint probabilistic modeling of single-cell multi-omic data with totalvi. *Nature methods*, 18:272 – 282, 2021.

[4] Yuhan Hao, Stephanie Hao, Erica Andersen-Nissen, William M. Mauck, Shiwei Zheng, Andrew Butler, Madeline J. Lee, Aaron J. Wilk, Charlotte A. Darby, Michael A. Zager, Paul J. Hoffman, Marlon Stoeckius, Efthymia Papalexi, Eleni P. Mimitou, Jaison Jain, Avi Srivastava, Tim Stuart,

Lamar M. Fleming, Bertrand Z. Yeung, Angela J. Rogers, Juliana M. McElrath, Catherine A. Blish, Raphael Gottardo, Peter Smibert, and Rahul Satija. Integrated analysis of multimodal single-cell data. *Cell*, 184:3573 – 3587.e29, 2021.

[5] Malte D. Luecken, Daniel B. Burkhardt, Robrecht Cannoodt, Christopher Lance, Aditi Agrawal, Hananeh Aliee, Ann Tai Chen, Louise Deconinck, Angela M. Detweiler, Alejandro A. Granados, Shelly Huynh, Laura Isacco, Yang Joon Kim, Bony De Kumar, Sunil Kuppasani, Heiko Lickert, Aaron McGeever, Honey E. Mekonen, Joaquín Caceres, Melgarejo, Maurizio Morri, Michael Mueller, Norma F. Neff, Sheryl Paul, Bastian, Rieck, Kaylie Schneider, Scott Steelman, Michael Sterr, Daniel J. Treacy, Alexander Tong, Alexandra-Chloé Villani, Guilin Wang, Jianrong Yan, Ce Zhang, Angela Oliveira Pisco, Smita, Krishnaswamy, Fabian J Theis, and Jonathan Michael Bloom. A sandbox for prediction and integration of dna, rna, and proteins in single cells. In *NeurIPS Datasets and Benchmarks*, 2021.

[6] Sai Ma, Bing Zhang, Lindsay M LaFave, Andrew S Earl, Zachary Chiang, Yan Hu, Jiarui Ding, Alison Brack, Vinay K Kartha, Tristan Tay, et al. Chromatin potential identified by shared single-cell profiling of rna and chromatin. *Cell*, 183(4):1103–1116, 2020.

[7] Xinjun Wang, Zhe Sun, Yanfu Zhang, Zhongli Xu, Hongyi Xin, Heng Huang, Richard H Duerr, Kong Chen, Ying Ding, and Wei Chen. Brem-sc: a bayesian random effects mixture model for joint clustering single cell multi-omics data. *Nucleic acids research*, 48(11):5814–5824, 2020.

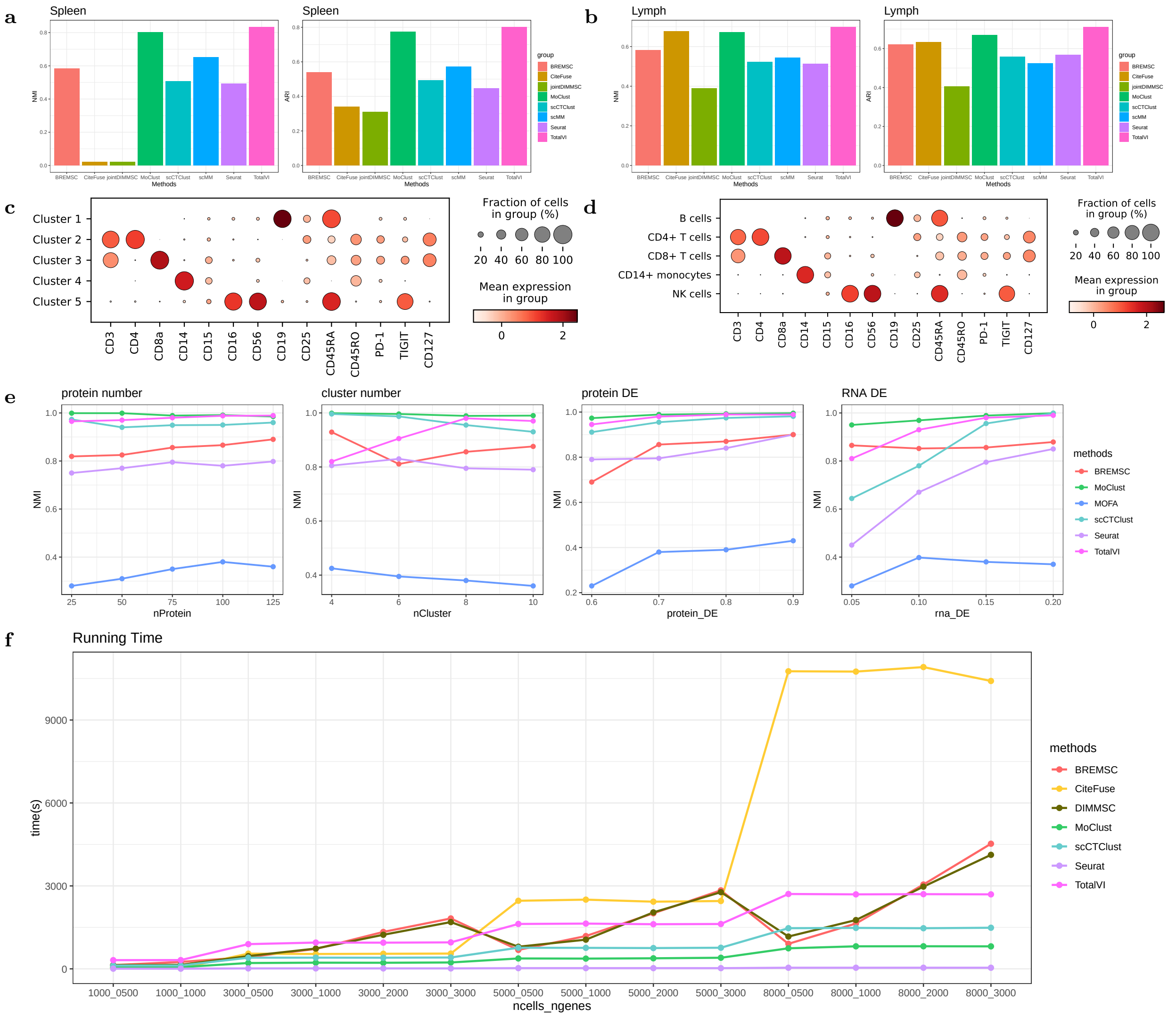

Figure 1: (a)&(b) The performance of MoClust and competing methods by NMI and ARI over real CITE-seq datasets Spleen and Lymph. (c)&(d) Dotplot of marker proteins against predicted clusters/true cell types, the scale of dot refers the fractions of cells in group while the color represents the mean expression. (e) CITE-seq simulation experiments. All simulated data were generated by Splatter and the performance of each method is evaluated by NMI. (f) The running time of MoClust and several methods over simulation datasets with different number of cells and features. '1000_0500' means 1000 cells each with 500 genes for an instance.

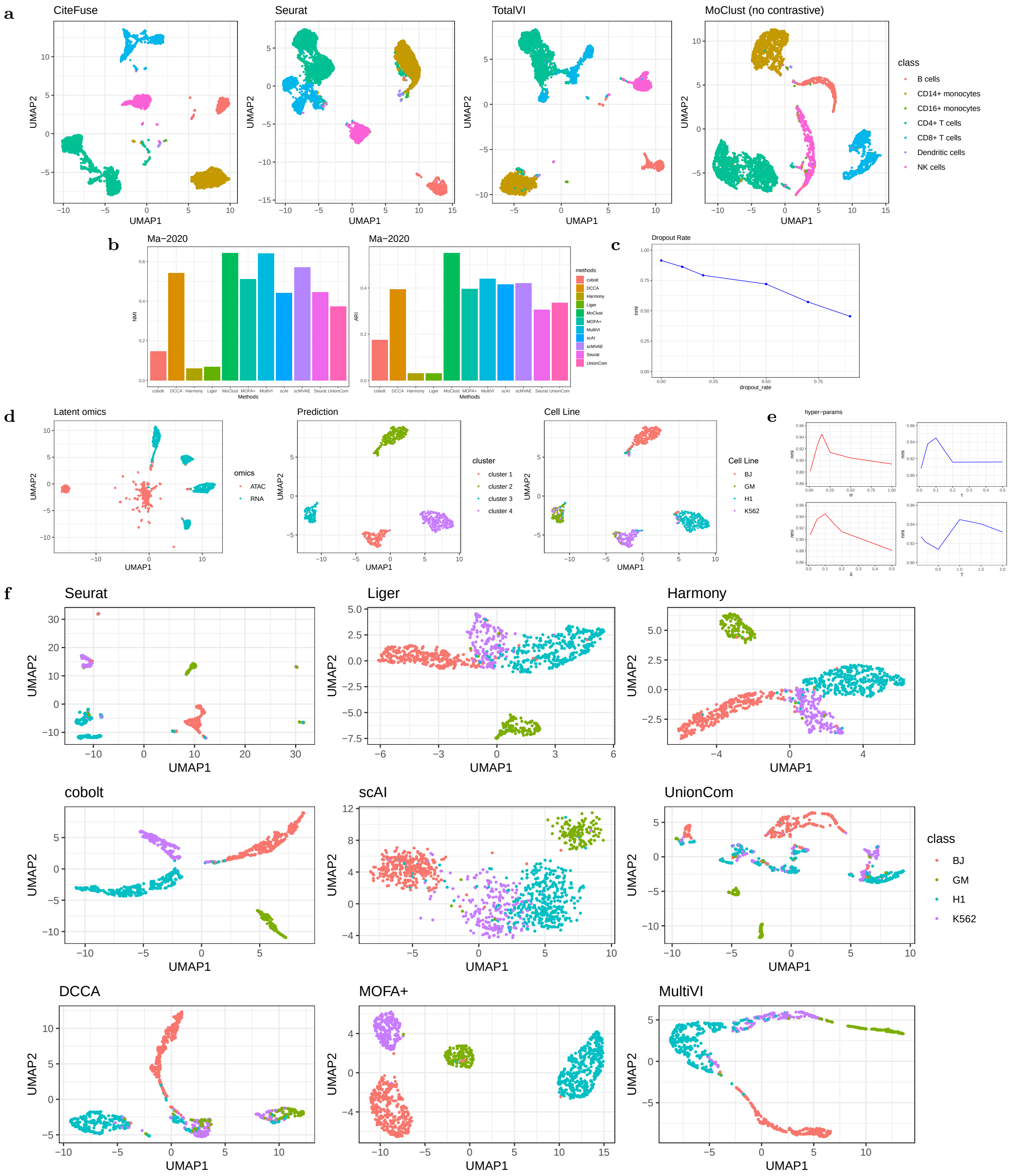

Figure 2: (a) UMAP visualization of latent features extracted by competing integrative methods, namely, CiteFuse, Seurat, TotalVI and MoClust without contrastive module, over the 10X10k dataset. (b) The performance of MoClust and competing methods by NMI and ARI over SHARE-seq dataset ma-2020. (c) The NMI of MoClust applying on 10XInHouse dataset with different dropout rate. (d) Two-dimensional visualization of latent features extracted by MoClust using the UMAP dimension reduction method. From left to right, the visualization of RNA and ATAC latent features, the fused features colored by predicted cluster and the fused features colored by true cell type are displayed. (e) Ablation study to determine the robustness of hyperparameters. (f) UMAP visualization of competing methods applying on CellLine dataset.

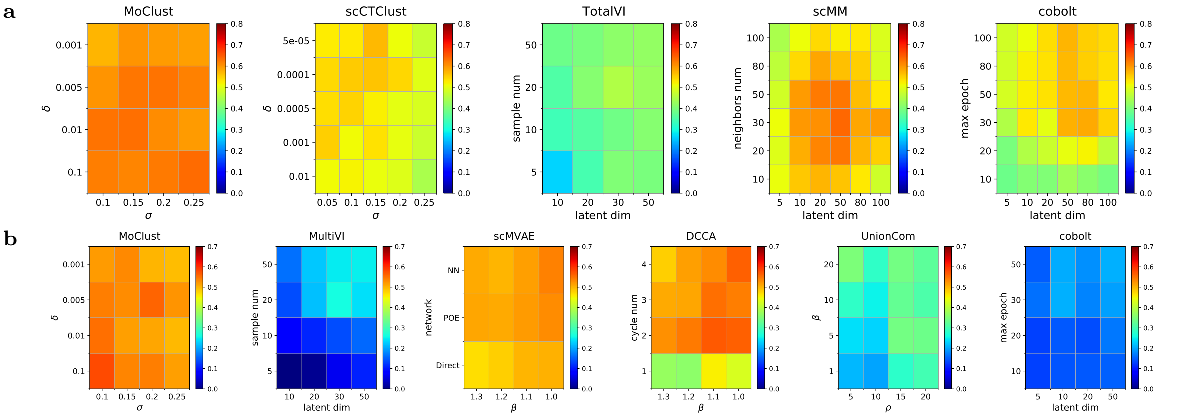

Figure 3: The results of deep learning methods evaluated by NMI under different hyper-parameters settings. (a) The performance of according methods applying on CITE-seq dataset BMMC-cite. (b) The performance of according methods applying on 10X multiome dataset BMMC-multiome.
